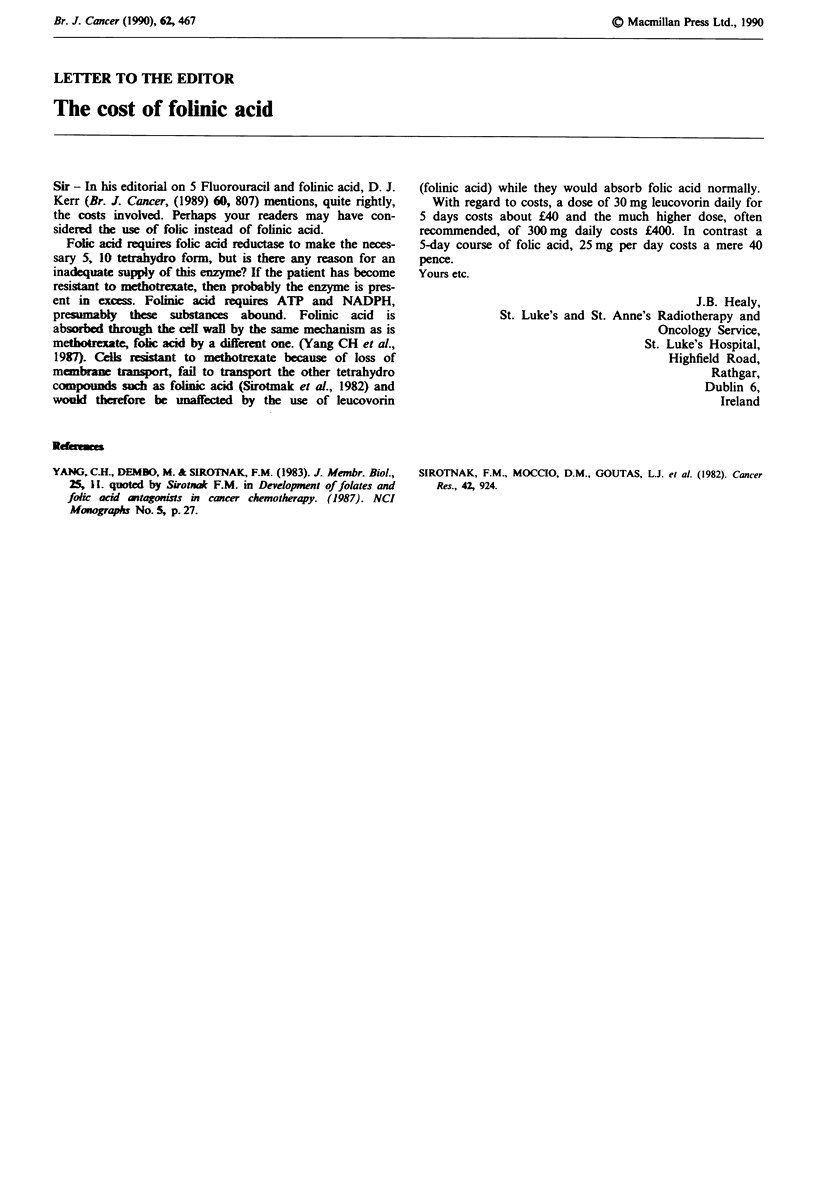# The cost of folinic acid.

**DOI:** 10.1038/bjc.1990.320

**Published:** 1990-09

**Authors:** J. B. Healy


 © Macmillan Press Ltd., 1990

LETTER TO THE EDITOR

### The cost of folinic acid

Sir – In his editorial on 5 Fluorouracil and folinic acid, D. J. Kerr (*Br. J. Cancer*, (1989) **60**, 807) mentions, quite rightly, the costs involved. Perhaps your readers may have considered the use of folic instead of folinic acid.

Folic acid requires folic acid reductase to make the necessary 5, 10 tetrahydro form, but is there any reason for an inadequate supply of this enzyme? If the patient has become resistant to methotrexate, then probably the enzyme is present in excess. Folinic acid requires ATP and NADPH, presumably these substances abound. Folinic acid is absorbed through the cell wall by the same mechanism as is methotrexate, folic acid by a different one. (Yang CH *et al.*, 1987). Cells resistant to methotrexate because of loss of membrane transport, fail to transport the other tetrahydro compounds such as folinic acid (Sirotmak *et al.*, 1982) and would therefore be unaffected by the use of leucovorin (folinic acid) while they would absorb folic acid normally.

With regard to costs, a dose of 30 mg leucovorin daily for 5 days costs about £40 and the much higher dose, often recommended, of 300 mg daily costs £400. In contrast a 5-day course of folic acid, 25 mg per day costs a mere 40 pence.

Yours etc.

J.B. Healy,
St. Luke's and St. Anne's Radiotherapy and Oncology Service,
St. Luke's Hospital,
Highfield Road,
Rathgar,
Dublin 6,
Ireland

### References

YANG, C.H., DEMBO, M. & SIROTNAK, F.M. (1983). *J. Membr. Biol.*, **25**, 11. quoted by *Sirotnak* F.M. in *Development of folates and folic acid antagonists in cancer chemotherapy. (1987). NCI Monographs* No. 5, p. 27.

SIROTNAK, F.M., MOCCIO, D.M., GOUTAS, L.J. *et al.* (1982). *Cancer Res.*, **42**, 924.